# Environmentally responsive genome-wide accumulation of de novo *Arabidopsis thaliana* mutations and epimutations

**DOI:** 10.1101/gr.177659.114

**Published:** 2014-11

**Authors:** Caifu Jiang, Aziz Mithani, Eric J. Belfield, Richard Mott, Laurence D. Hurst, Nicholas P. Harberd

**Affiliations:** 1 State Key Laboratory of Plant Physiology and Biochemistry, College of Biological Sciences, China Agricultural University, Beijing 100094, China;; 2 Department of Plant Sciences, University of Oxford, Oxford OX1 3RB, United Kingdom;; 3 Department of Biology, Syed Babar Ali School of Science and Engineering, Lahore University of Management Sciences (LUMS), DHA, Lahore 54792, Pakistan;; 4 Wellcome Trust Centre for Human Genetics, University of Oxford, Oxford OX3 7BN, United Kingdom;; 5 Department of Biology and Biochemistry, University of Bath, Bath BA2 7AY, United Kingdom

## Abstract

Evolution is fueled by phenotypic diversity, which is in turn due to underlying heritable genetic (and potentially epigenetic) variation. While environmental factors are well known to influence the accumulation of novel variation in microorganisms and human cancer cells, the extent to which the natural environment influences the accumulation of novel variation in plants is relatively unknown. Here we use whole-genome and whole-methylome sequencing to test if a specific environmental stress (high-salinity soil) changes the frequency and molecular profile of accumulated mutations and epimutations (changes in cytosine methylation status) in mutation accumulation (MA) lineages of *Arabidopsis thaliana*. We first show that stressed lineages accumulate ∼100% more mutations, and that these mutations exhibit a distinctive molecular mutational spectrum (specific increases in relative frequency of transversion and insertion/deletion [indel] mutations). We next show that stressed lineages accumulate ∼45% more differentially methylated cytosine positions (DMPs) at CG sites (CG-DMPs) than controls, and also show that while many (∼75%) of these CG-DMPs are inherited, some can be lost in subsequent generations. Finally, we show that stress-associated CG-DMPs arise more frequently in genic than in nongenic regions of the genome. We suggest that commonly encountered natural environmental stresses can accelerate the accumulation and change the profiles of novel inherited variants in plants. Our findings are significant because stress exposure is common among plants in the wild, and they suggest that environmental factors may significantly alter the rates and patterns of incidence of the inherited novel variants that fuel plant evolution.

In *The Origin of Species*, Darwin identified heritable variation as fundamental to biological evolution (Darwin 1859), although he could not define that variation. We now understand that the heritable variation underlying evolution is substantially due to genetic (e.g., DNA sequence mutation) and potentially to epigenetic (e.g., altered cytosine methylation or histone modification status) change (Mitchell-Olds and Schmitt 2006; Baer et al. 2007; Nordborg and Weigel 2008; Richards 2008; Atwell et al. 2010; Liu et al. 2010; Schmitz et al. 2011, 2013; Schmitz and Ecker 2012). Furthermore, recent advances have provided a provisional genome-wide picture of how genetic and epigenetic changes accumulate during successive generations. For example, previous studies have characterized the de novo variants accumulating in mutation accumulation (MA) lineages of the genetic model plant *Arabidopsis thaliana* (Ossowski et al. 2010; Becker et al. 2011; Schmitz et al. 2011). These studies have revealed the frequencies and patterns with which mutations and epimutations accumulate in MA lineages grown in relatively sheltered artificial laboratory environments. However, the natural environment is rarely as benign as these laboratory environments, and plants growing in nature are frequently exposed to varying combinations of environmental stresses (Easterling et al. 2000; Parmesan and Yohe 2003; Mittler 2006). Furthermore, the phenomenon of stress-induced mutagenesis (SIM), in which mutation is promoted when cells are poorly adapted to their environment, is well established in bacteria (Al Mamun et al. 2012) and more recently identified in yeast and human cancer cells (Bindra et al. 2007; Shor et al. 2013). We therefore sought to determine if propagation of *A. thaliana* MA lineages in a stressful environment changes the rates and profiles of de novo variant accumulation, reasoning that such changes could have important implications for the understanding of plant genome evolution in nature.

Soil salinity is a widespread source of plant abiotic stress, affecting ∼6% of global land area (Hasegawa et al. 2000; Zhu 2002; Munns and Tester 2008). Plants have evolved mechanisms to circumvent the effects of high soil Na^+^, the most prevalent ionic form of natural soil-salinity (Zhu 2002). While the short-term physiological consequences of exposure to high soil-salinity are increasingly well understood (Rus et al. 2001; Zhu 2002; Ren et al. 2005; Munns and Tester 2008; Baxter et al. 2010; Jiang et al. 2012, 2013; Zhou et al. 2012), the longer-term evolutionary genetic and epigenetic consequences are not. For example, it was not previously known if multiple successive generations of exposure to soil-salinity stress changes the properties of genome-wide accumulated de novo variants, thus in turn affecting evolutionary processes. Here we directly address this issue and show that *A. thaliana* (The *Arabidopsis* Genome Initiative 2000) MA lineages grown for 10 successive generations on saline soil display an increased frequency of accumulated de novo mutations and epimutations (differentially methylated cytosine positions, DMPs). We also show that the mutations accumulating during soil-salinity stress exhibit a distinctive molecular mutational spectrum that differs from that of mutations accumulating in nonstressed control MA lineages. Our observations have important implications for the understanding of plant genome evolution in the stressful natural environment.

## Results

### The establishment of saline soil-grown mutation accumulation (MA) lineages

**Figure 1.**
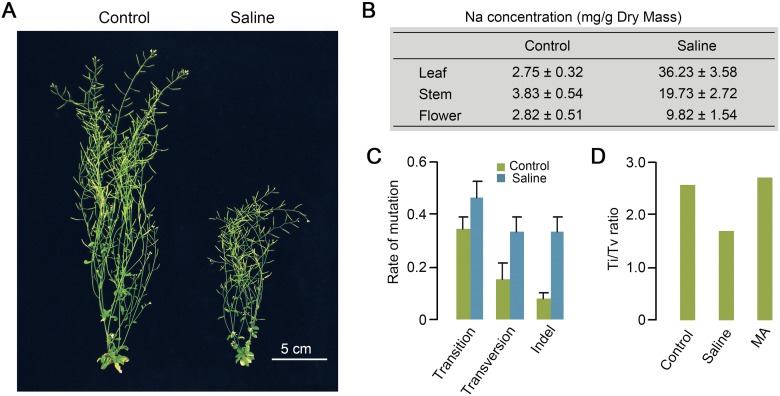
The morphological, physiological, and mutational effects of saline soil on *A. thaliana*. (*A*) Retarded growth of 10-wk-old *A. thaliana* (Col-0) plants growing on Saline (versus Control) soil. (*B*) Sodium (Na) content in rosette leaves (Leaf), inflorescence stems (Stem), and flowers with immature siliques (Flower) of four 8-wk-old plants grown in Control or Saline soil. Results are triplicate measurements of two biological replicates. (*C*) Rates (per genome per generation) of Transition, Transversion, and Indel (1–226 bp) (Supplemental Table S3) mutations accumulated in lineages grown for 10 successive generations on Saline or Control soil. Error bars represent standard error of the mean. (*D*) Transition/Transversion ratios (Ti/Tv) of single base substitutions accumulated in lineages grown for 10 successive generations on Control or Saline soil. For comparison, the Ti/Tv ratio characteristic of mutations accumulating in Control soil in a previous experiment (MA) is also shown (Ossowski et al. 2010).

We established *Arabidopsis thaliana* mutation accumulation (MA) lineages (derived from the same Col-0 laboratory strain progenitor plant) on either control or saline soil (see Methods; Supplemental Fig. S1). The salinity of the saline soil was sufficient to cause pronounced stress symptoms (e.g., growth retardation) (Fig. 1A), elevated tissue Na content throughout the shoots of exposed plants (Fig. 1B), and prolonged generation times (from ∼9 to ∼12 wk) (data not shown). Nevertheless, this salinity level was not sufficient to prevent sexual propagation, and, in total, we propagated six independent MA lineages for 10 successive generations on control (three lineages) and saline (three lineages) soil (see Methods; Supplemental Fig. S1). We subsequently identified the de novo genetic and epigenetic variants accumulated in 10^th^ generation (G10) plants from each MA lineage (see Methods). Our isogenic experimental design permitted the accurate calling of de novo genetic and epigenetic variants and enabled the observations described in depth in subsequent sections of this paper.

### De novo DNA sequence mutations accumulate with increased frequency and a distinctive molecular mutational spectrum in saline soil-grown MA lineages

We first determined if propagation for multiple successive generations in a saline soil environment alters the genome-wide frequency and spectrum of accumulated DNA sequence mutations in *A. thaliana*. Whole-genome sequencing (Illumina 90-bp paired-end reads; 20–27× genome coverage) (Supplemental Table S1; see Methods) of three individual G10 plants from each of the six control and saline MA lineages (and of a single G0 plant) (Supplemental Fig. S1) enabled identification (by comparison with the G0 sequence) of 102 de novo homozygous DNA sequence mutations in G10 saline soil plants (versus 52 in G10 controls) (Supplemental Tables S2, S3; see Methods). Most identified de novo mutations were single base substitutions (SBSs) or short (1- to 3-bp) indels (insertions/deletions) (Supplemental Table S2; Supplemental Fig. S2). Growth on saline soil caused an approximately twofold increase in overall mutation rate (*t*-test, *P* = 2 × 10^−4^) (Supplemental Table S2) and increased the incidence of specific mutational classes: Transversions (*t*-test, *P* = 0.05) and indels (*t*-test, *P* = 6 × 10^−4^) were significantly increased in frequency, while transitions were not (*P* = 0.12) (Fig. 1C). We found that 30 of the 44 de novo SBSs in our control soil MA lineages were transitions (Supplemental Table S2), giving a transition/transversion (Ti/Tv) ratio of 2.48 (Fig. 1D). This transition predominance is in accord with previous observations of laboratory-grown *A. thaliana* MA lineages (2.73) (Fig. 1D; Ossowski et al. 2010), indicating that a Ti/Tv ratio of ∼2.5 is characteristic of SBSs accumulating in Col-0 *A. thaliana* plants grown in standard laboratory conditions. In contrast, ∼43% (31 out of 72) (Supplemental Table S2) of the SBSs identified in G10 saline soil plants were transversions, resulting in a significantly depressed Ti/Tv ratio of 1.70 (Fisher’s exact test, *P* = 0.009) (Fig. 1D). These observations suggest that multigenerational growth of *A. thaliana* in saline soil increases the frequency and changes the molecular mutational spectrum of accumulated de novo DNA sequence mutations. The possible evolutionary consequences of these findings are considered in the Discussion.

### Multigenerational propagation of MA lineages on saline soil has little effect on overall genome-wide cytosine methylation pattern

We next turned our attention to the accumulation of genome-wide epigenetic change (change in cytosine methylation status) in control and saline soil MA lineages. We first determined if saline soil MA lineages accumulate overall genome-wide cytosine methylation changes that detectably distinguish them from control MA lineages. We performed whole-genome bisulfite sequencing (Illumina 90-bp paired-end reads; 70–88× genome coverage) (Supplemental Table S4; see Methods) on the genomes of single G10 plants from each of the saline soil (G10-S1, -S2, and -S3) and control (G10-C1, -C2, and -C3) MA lineages and on two single first-generation plants (G1-1 and -2) (Supplemental Fig. S3). Importantly, in order to minimize the detection of transient epigenetic change elicited by direct exposure to stress (Dowen et al. 2012), the sequenced G10 saline soil MA lineage plants were actually grown in control (rather than saline) soil (Supplemental Fig. S3). Of all cytosine residues covered, at least threefold with high-quality sequencing reads (see Methods), on average, ∼3.9 million were found to be methylated in each line (Supplemental Table S5; see Methods for criteria used to call cytosine methylation status).

We next compared the genome-wide DNA methylation patterns in genomes of G10 plants from control and saline soil MA lineages by evaluating the ∼38.2 million cytosines that had ≥threefold and ≤200-fold coverage in all G10 and G1 samples, of which ∼5.2 million were detectably methylated in at least one sample (Supplemental Table S6A). Cytosine residues may be methylated within any of three distinct sequence contexts (CG, CHG, and CHH, where H is A, T, or C) via the action of cytosine methyltransferase enzymes (Cao and Jacobsen 2002; Kankel et al. 2003; Chan et al. 2005). Consistent with previous reports (Zhang et al. 2006; Cokus et al. 2008; Becker et al. 2011), we found cytosines within centromere regions, pseudogenes, and transposable elements (TEs) to be particularly prone to methylation (Supplemental Fig. S4). In addition, G10 saline soil and control plants displayed relatively similar genome-wide DNA methylation distribution patterns (Supplemental Fig. S5), indicating that long-term exposure to soil-salinity does not cause obvious gross change (increase or decrease) in genome-wide cytosine methylation.

### Multigenerational propagation of MA lineages on saline soil promotes accumulation of de novo differentially methylated CG positions (CG-DMPs)

**Figure 2.**
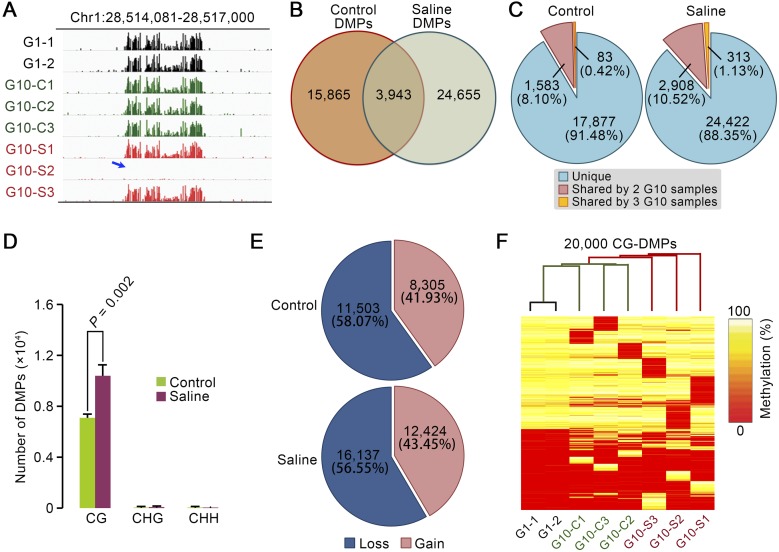
Multigenerational propagation on saline soil increases the accumulation of differentially methylated CG positions (CG-DMPs) in MA lineages. (*A*) IGV (Integrated Genome Viewer) (see Methods) views of methylation level differentials. Column height indicates the relative extent of methylation at individual cytosine positions in G1 (black), saline soil G10 (red), and control G10 (green) genome samples on a specific region of chromosome 1. The blue arrow indicates a cluster of differentially methylated cytosine positions (DMPs) where methylation has been lost in sample G10-S2. (*B*) Number and overlap of DMPs detected in G10 control (Control DMPs) and saline soil (Saline DMPs) samples. (*C*) Frequency of DMP sharing within control (Control) versus saline soil (Saline) treatments. “Shared” refers to DMPs shared within (rather than between) treatments (Control or Saline). (*D*) Comparison of the number of DMPs at CG, CHG, and CHH sites in G10 saline soil (Saline) and control samples. Results shown are means ±SD. (*E*) Percentages of DMPs due to loss or gain of methylation in G10 samples. Data from each group (Control or Saline soil) were combined. (*F*) Hierarchical clustering of samples based on selected sets of 20,000 sites drawn randomly from CG-DMPs identified in all samples.

We next turned our attention to individual cytosine positions where methylation status had changed (through gain or loss of methylation) during propagation of the MA lineages by comparing the number of de novo DMPs in the genomes of saline soil and control G10 plants. Using Fisher’s exact test, we characterized positions displaying a significant change in methylation status (false discovery rate <0.05) (see Methods) in at least one of the G10 plants (i.e., a methylation status that differed from the status observed in both G1 plants) (examples of DMPs are shown in Fig. 2A). A total of 28,598 DMPs were identified in at least one of the saline soil G10 samples, versus 19,808 in at least one of the control G10 samples. Because 3943 DMPs were shared between saline soil and control G10 samples, 24,655 DMPs were unique to saline soil G10 samples and 15,865 were unique to control G10 samples (Fig. 2B; Supplemental Table S6B). Further exploration of the properties of the DMPs accumulated in saline soil and control G10 samples revealed that ∼90% of CG-DMPs were identified only in a single lineage (i.e., were not shared between one or more lineages within treatment [control or saline soil]) (Fig. 2C; Supplemental Table S7). It is worth noting that our DMP numbers may be an underestimate, because some DMPs might not have been detected (Laird 2010; Bock 2012). However, because bioinformatics analyses of our control and saline soil-grown plant data sets were performed in the same way, false negative rates will be similar in all data sets, and will therefore have a negligible effect on the comparative conclusions that we draw.

As in a previous study (Becker et al. 2011), we found DMPs at CG sites (CG-DMPs) to be highly overrepresented among total DMPs in both control and saline soil G10 samples. Note that this overrepresentation likely reflects a greater power of detection of change at CG sites (Fig. 2D; Becker et al. 2011). Most notably, we found that the saline soil G10 samples displayed ∼45% more CG-DMPs than controls (*t*-test, *P* = 0.002) (Fig. 2D), with no apparent preference for decrease (loss) or increase (gain) of methylation (Fig. 2E). These results suggest that multigenerational growth on saline soil increases the frequency of accumulation of CG-DMPs. This conclusion is further supported by hierarchical clustering analysis of randomly selected CG-DMPs, which showed that the G1 (progenitor) and G10 control samples grouped together, and that the G10 saline soil samples were more diverged from this grouping (Fig. 2F).

### Multigenerational propagation of MA lineages on saline soil promotes accumulation of regionally clustered de novo differentially methylated CG positions (CG-DMRs)

**Figure 3.**
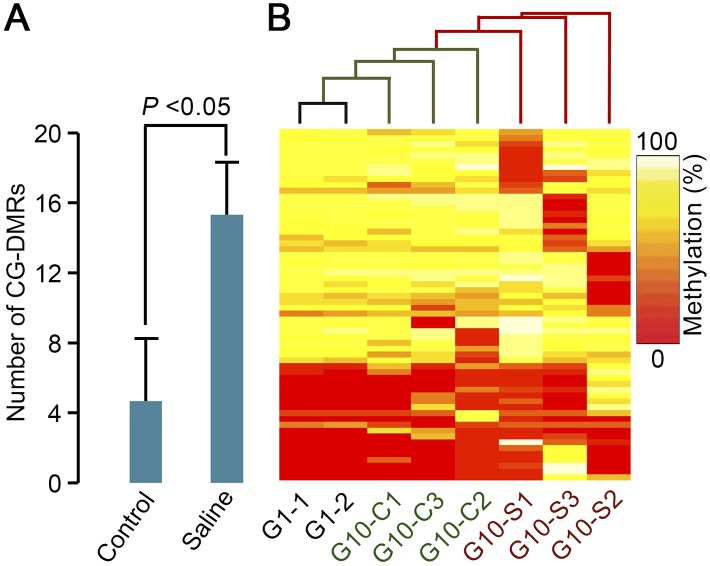
Multigenerational propagation on saline soil increases the accumulation of regional clusters of differentially methylated CG positions (CG-DMRs). (*A*) Comparison of the number of regional clusters of differentially methylated CG positions (CG-DMRs) (as defined in Methods) identified in saline soil (Saline) and control G10 plant genome samples. Results shown are means ±SD. (*B*) Hierarchical clustering of samples based on identified CG-DMRs.

Clustered change in DNA methylation status in specific genomic regions (in differentially methylated regions [DMRs] i.e., genomic regions with clustered DMPs) (see Methods) can influence gene activity by various mechanisms (e.g., alteration of mRNA transcript levels via change in affinity of transcription factors for gene promoter sequences or RNA splicing) (Jacobsen and Meyerowitz 1997; Zilberman et al. 2007; Gelfman et al. 2013). We determined if G10 saline soil genomic samples contained more DMRs with respect to differentially methylated CG positions (CG-DMRs) (see Methods) than controls. We identified 46 CG-DMRs in saline soil G10 samples and 14 in controls (see Methods). Although the occurrence of DMRs is relatively infrequent, the mean frequency of accumulated CG-DMRs in saline soil MA lineages was increased ∼200% over that of control lineages (Fig. 3A). The mean DMR size was ∼40 bp in control and ∼50 bp in saline soil lineages, with the majority of DMRs (11 out of 14 in G10 control and 34 out of 46 in G10 saline soil samples) occurring in genic regions (Supplemental Table S8). Hierarchical clustering based on methylated sites in regions identified as CG-DMRs in G10 individuals indicated that saline soil lineage G10 genomes were more divergent from G1 genomes than were control lineage G10 genomes (Fig. 3B). We conclude that *A. thaliana* lineages growing in saline soil conditions accumulate more CG-DMRs than do lineages growing in control conditions.

### CG-DMPs and CG-DMRs accumulated in MA lineages are frequently retained in subsequent generations

**Figure 4.**
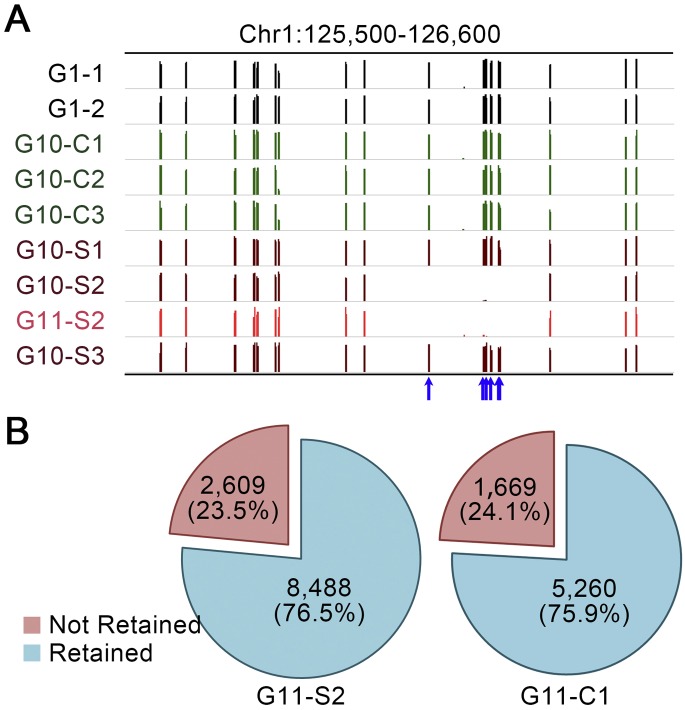
Stability of inheritance of CG-DMPs accumulated during multigenerational propagation on saline soil. Comparisons of methylation status at CG-DMPs in genomes of G10 and G11 plants. (*A*) IGV views of methylation level differentials. Column height indicates the relative extent of methylation at individual CG positions in G1 (black), control G10 (green), saline soil G10 (dark red), and saline soil G11 (red) genome samples in a specific region of chromosome 1. The blue arrows highlight example CG-DMPs where methylation has been lost in one of the saline soil G10 samples (G10-S2). Absence of methylation at these CG-DMPs is retained (stably inherited) in the subsequent generation, in the G11-S2 genome sample (see Supplemental Fig. S3). (*B*) Overall frequencies of retention of methylation status in G11 plants. CG-DMPs identified in G10 plants (G10-S2 or G10-C1) are shown as Retained (unchanged methylation status [stably inherited] in G11-S2 or G11-C2) or as Not Retained (where methylation status differs between G10 and G11 genomes).

Because altered cytosine methylation status is potentially unstable (relative to DNA sequence mutation), we next assessed the stability of methylation status at CG-DMPs accumulated in both control and saline MA lineages. Plant G11-C1 was a self-pollination-derived offspring of plant G10-C1 (Supplemental Fig. S3) and thus representative of a history of multigenerational growth on control soil. Plant G11-S2 was a self-pollination-derived offspring of plant G10-S2, and, being itself grown on control soil, represented a history of multigenerational growth on saline soil (10 successive generations) with two subsequent generations on control soil (Supplemental Fig. S3). We analyzed the methylation status of the genome of G11-C1 and G11-S2 at those cytosine positions where CG-DMPs had previously been identified in the genomes of plants G10-C1 or G10-S2 (i.e., in the preceding generation). Figure 4A highlights example CG-DMPs where methylation had been lost from the genome of plant G10-S2 (but not from the genomes of G10 plants representative of the other MA lineages). Methylation status (absence of methylation) at these highlighted CG-DMPs was stably inherited by plant G11-S2 (Fig. 4A), showing that, in these particular cases, a change in methylation status accumulated during multigenerational growth on saline soil was stably inherited over two subsequent generations of growth on control soil. Assessing all of the CG-DMPs previously identified in the genomes of plants G10-S2 or G10-C1, we found that ∼76.5% (8488 out of 11,097) of G10-S2 CG-DMPs retained their methylation status in G11-S2 and that ∼75.9% (5260 out of 6929) of G10-C1 CG-DMPs retained their methylation status in G11-C1 (Fig. 4B). Although based on only single G11-C1 and G11-S2 plants, these observations suggest that the majority of the changes in cytosine methylation status accumulated in both control and saline MA lineages are retained in subsequent generations. However, these findings also suggest that there is significant loss of methylation status of CG-DMPs in subsequent generations.

### Salinity-associated CG-DMPs arise more frequently in genic than in nongenic regions of the genome

**Figure 5.**
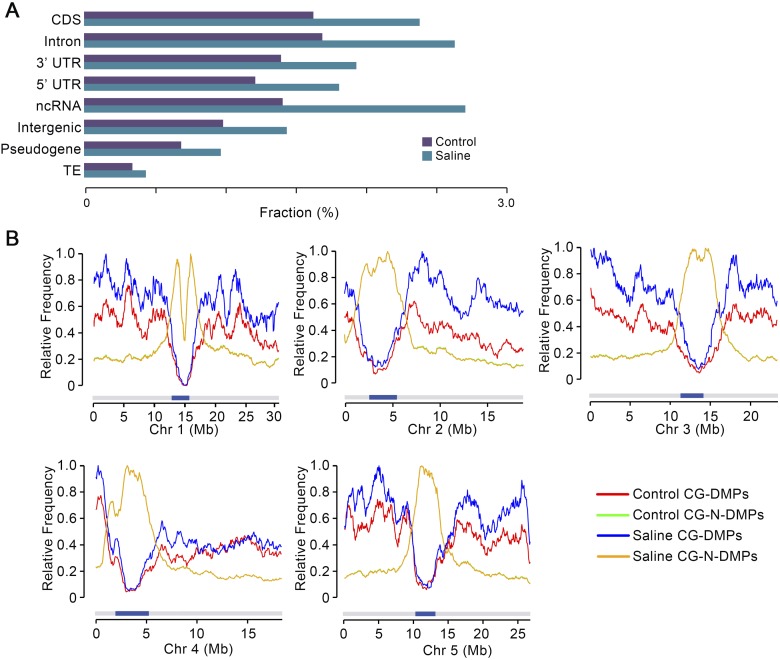
Genome-wide distribution of CG-DMPs in saline soil and control MA lineages. (*A*) Distribution of CG-DMPs in genomic regional categories as determined by genome sequence annotation. (CDS) Coding DNA sequence; (UTR) untranslated region; (ncRNA) noncoding RNA; (TE) transposable element, expressed as fraction of total methylated GC sites in all saline soil and control G10 samples. (*B*) Distribution along individual chromosomes (Chr 1, Chr 2, etc.) of CG-DMPs and CG-N-DMPs (see text for definition of CG-N-DMPs). Data from G10 saline soil and control samples were normalized to the highest value for each chromosome and class (CG-DMPs or CG-N-DMPs). Dark blue bars indicate centromeres. Because Control and Saline CG-N-DMPs track very closely, the latter trace substantially obscures the former.

Further exploration of the properties of the DMPs accumulated in saline soil and control MA G10 samples showed that genic (CDS, intron, and UTR) and ncRNA genomic regions display a relatively higher frequency of CG-DMPs than do nongenic (intergenic, pseudogene, and TE) regions (Fig. 5A; see also Becker et al. 2011). In addition, while there was an overall increase (versus control G10 samples) in CG-DMP frequency in all genomic regions, the genic and ncRNA regions showed a greater increase than nongenic regions in G10 saline samples (*G*-test, *P* = 1.65 × 10^−141^) (Fig. 5A). Consistent with these observations, CG-DMPs, but not CG-N-DMPs (CG positions where methylation status remained unchanged), were mostly located on chromosome arms (where gene density is higher than in the centromeres) in both saline soil and control G10 samples (Fig. 5B), with the increased numbers of CG-DMPs occurring in saline soil G10 samples also being largely chromosome arm located (Fig. 5B). These observations may be reflective of a differential propensity for change in cytosine methylation status of DNA in different chromatin states (e.g., open versus closed chromatin).

## Discussion

### Multigenerational exposure of *A. thaliana* to soil salinity stress accelerates the genome-wide accumulation of mutations and epimutations

Using whole-genome sequencing approaches, we have shown that *A. thaliana* MA lineages propagated for multiple successive generations on saline soil accumulate more DNA sequence mutations and epimutations (changes in cytosine methylation status) than controls. However, the extent to which this increased accumulation is passive (an incidental consequence of stress exposure) versus active (an organismally regulated response to environmental stress) is not currently clear (see below for further discussion). In some cases, organisms exposed to environmental stress actively alter the rates and patterns with which de novo genetic (and possibly epigenetic) variants arise, in turn potentially promoting long-term evolutionary adaptation (Baer et al. 2007; Bindra et al. 2007; Rando and Verstrepen 2007; Chinnusamy and Zhu 2009; Lynch 2010; Al Mamun et al. 2012; Shor et al. 2013). This concept is exemplified by the well-known bacterial SOS response mechanism (Bjedov et al. 2003; Baer et al. 2007; Rando and Verstrepen 2007) and by related SIM mechanisms (Baer et al. 2007; Bindra et al. 2007; Al Mamun et al. 2012; Shor et al. 2013). However, it is also the case that increased rates of accumulation of mutation and/or epimutation will increase the load of deleterious mutations.

### The de novo variants accumulated following multigenerational exposure of *A. thaliana* to soil salinity stress display distinct molecular profiles

The increased accumulation of genetic/epigenetic variants seen in plants exposed to stressful saline soil conditions is not simply explained by a general increase in all classes of mutation/epimutation. We have shown that multigenerational growth of *A. thaliana* on saline soil promotes significant increases in the rate of accumulation of specific classes of both de novo DNA sequence mutations and epimutations (changes in cytosine methylation status). First, the rate of accumulation of de novo transversion and indel mutations is significantly increased, while that of de novo transition mutations is not (Fig. 1C). The transition/transversion (Ti/Tv) ratio of single base substitutions accumulating in *A. thaliana* MA lineages exposed over multiple successive generations to soil-salinity stress (∼1.7) is different from that of control MA lineages (∼2.5) (Fig. 1C), but similar to that of mutations accumulated in in vitro regenerant or irradiated *A. thaliana* lineages (Jiang et al. 2011; Belfield et al. 2012). Intriguingly, the change in Ti/Tv ratio generated by salt stress in *A. thaliana* is similar to that generated by the environmental stress response in yeast (Shor et al. 2013). Furthermore, the ∼1.7 Ti/Tv ratio is comparable with that of natural variant SNPs (∼1.6) (Cao et al. 2011), suggesting that environmental factors (e.g., stresses) may substantially affect the molecular spectrum of mutations arising de novo in *Arabidopsis* lineages growing in nature.

Second, we have shown that multigenerational exposure of *A. thaliana* to soil-salinity stress is associated with a significant increase in the rate of accumulation of CG-DMPs (Fig. 2C). While many of these CG-DMPs are stably inherited in subsequent generations, there is also significant generation-by-generation loss (Fig. 4), observations comparable with those of previous studies (e.g., Becker et al. 2011; Schmitz et al. 2011, 2013; Stroud et al. 2013). Notably, the observed increase in CG-DMP accumulation following multigenerational exposure to soil-salinity stress is particularly prominent in genic regions of the genome, and in many cases is clustered in the form of DMRs (Figs. 2D, 3). Thus multigenerational exposure to environmental stress changes the genome-wide molecular profiles of mutations and epimutations accumulating in *A. thaliana* MA lineages.

### How might soil-salinity stress change the rate and pattern of mutation acquisition in *A. thaliana*?

Three main hypotheses have been proposed to account for mutational variation between lineages (Baer et al. 2007), and these hypotheses potentially also explain stress-associated variation. First, the “generation-time” hypothesis is based on the idea that mutations arise due to DNA replication errors and suggests that organisms with shorter generation times accumulate mutations faster because they go through more rounds of (stem cell) cell divisions (and hence DNA replication) during an arbitrary unit of time. In the case of our saline soil lineages, the generation time is longer than that of control lineages. We do not know if this is associated with an increased number of cell divisions per generation, or if the cell cycle is slowed, with longer generation times equating to the same number of longer cell cycles. It therefore remains possible that the generation-time hypothesis might account for at least some of the change in rate and pattern of mutations acquired during growth of *A. thaliana* on saline soil.

Second, the “metabolic” hypothesis suggests, in particular, that increased amounts of free radicals (Baer et al. 2007) such as reactive oxygen species (ROS) lead to increased DNA damage and hence faster rates of mutation. This hypothesis may well account for some of the increased mutations seen in *A. thaliana* grown on saline soil, because salinity stress is well known to increase ROS levels in plants (Hasegawa et al. 2000; Zhu 2002; Mittler 2006; Munns and Tester 2008).

Finally, the “DNA repair” hypothesis suggests that variation in fidelity of DNA repair may account for variation in mutation rate (Baer et al. 2007). Possible mechanisms for alteration in DNA repair in *A. thaliana* plants grown on saline soils include stress-related impairment of DNA repair activity or potential up-regulation of the error-prone polymerases typical of the SOS and SIM mechanisms identified in bacteria, yeast, and human cancer cells (Baer et al. 2007; Bindra et al. 2007; Rando and Verstrepen 2007; Al Mamun et al. 2012; Shor et al. 2013).

## Conclusions and context

Our conclusions have the following broader evolutionary consequences. First, our demonstration that environmental stress accelerates the accumulation of mutations and epimutations in *A. thaliana* lineages suggests that the rates and patterns of accumulation of novel variants observed in nonstressed, laboratory-grown MA lineages may not accurately reflect novel variants accumulating in nature. This is an important consideration, because a full understanding of biological evolution requires accurate knowledge of the de novo heritable variation that fuels it.

Second, the increased frequency of de novo mutation characteristic of stressful environments is likely to increase the accumulation of deleterious mutations, and might in some cases lead to accelerated population decline. Plant populations growing in natural environments are therefore likely to carry a greater load of deleterious de novo mutations than populations grown in optimal laboratory environments.

Third, our demonstration that environmental stress alters the properties of accumulating de novo variants could impact the evolution of gene function. An increased rate of transversion accumulation would be expected to accelerate protein evolution (Zhang 2000) (especially in species with low effective population size), not least by increasing the proportion and number of mutations that are nonsynonymous at twofold degenerate codons. In addition, an increased rate of accumulated change of genic GC methylation status might promote evolution of gene regulation (transcription) (Jacobsen and Meyerowitz 1997; Zilberman et al. 2007; Gelfman et al. 2013), although the extent to which this might contribute to the evolution of gene function remains unclear, given that we also observe a significant rate of loss of change of methylation status.

Finally, our observations likely relate to natural evolution. Plants living in the wild encounter substantial potential and actual environmental stress challenges throughout their life cycles. Our findings suggest that exposure to these challenges alters the rates and patterns of incidence of inherited de novo variants in plants. Similarly, environmental and physiological stress affects mutational processes in *Drosophila* and *Caenorhabditis* species (Agrawal and Wang 2008; Matsuba et al. 2013; Sharp and Agrawal 2013). Thus, environmental factors may affect rates and patterns of incidence of the novel genetic variation that fuels biological evolution in nature.

## Methods

### Plant materials and growth conditions

The Col-0 laboratory strain of *A. thaliana* used in this study was as previously described (Jiang et al. 2011). Plants were grown in a long-day (16 h light/8 h dark) photoperiod (light irradiance 120 μmol photons m^−2^ s^−1^) at 22–24°C.

### Propagation of control and saline soil *A. thaliana* mutation accumulation lineages

A single ancestral Col-0 plant was self-pollinated, and ∼600 progeny seed were divided into six groups of ∼100 seeds each. The groups were planted in soil, three groups in soil saturated with 125 mM NaCl (Saline soil) and three groups in control, water-saturated soil (Control) (Supplemental Fig. S1). Following 3 d of stratification at 4°C, the six seed groups were transferred to a glasshouse (conditions as described above). The resultant “Generation 0” (Supplemental Fig. S1) plants (both Control and Saline soil groups) were then grown to adulthood (and watered periodically without the addition of further NaCl), and allowed to self-pollinate. Seed was collected, and ∼200 “Generation 1” seeds were then sown from each lineage. This process was repeated for nine subsequent generations, resulting in six independent mutation accumulation lineages of 10 generations span each (Supplemental Figs. S1, S3).

### Salt content determination

Plant material was oven-dried at 80°C, weighed, and then digested in concentrated (69% v/v) HNO_3_ for at least 12 h. Sodium concentrations were determined in appropriately diluted samples using an atomic absorption spectrophotometer (Analysis100, Perkin-Elmer) as previously described (Jiang et al. 2012, 2013).

### Preparation of DNA samples for whole-genome sequencing and whole-genome bisulfite sequencing

For whole-genome sequencing, rosette leaves were taken from single G10 (Generation 10) MA lineage plants and from a G0 progenitor plant (Supplemental Fig. S1). DNA was isolated using a Plant DNeasy Mini kit (Qiagen). For whole-genome individual plant bisulfite sequencing, rosette leaf samples were taken from single G10 plants, from plants G11-C1 and G11-S2, and from two G1 progenitor plants (Supplemental Fig. S3); all plants were grown in control (nonsaline) conditions, and DNA samples were prepared as described above.

### Whole-genome DNA sequencing, sequence alignment, and variant calling

Genomic DNA samples were sequenced using standard Illumina 90-bp paired-end sequencing technology at BGI, China. Sequencing data sets were mapped to the TAIR10 reference genome using Stampy (Lunter and Goodson 2011), followed by five iterative mappings of unmapped reads using IMR/DENOM (Gan et al. 2011). Only high-quality (*phred* score ≥20) and uniquely mapped reads were used for variant detection (uniquely mapped read coverage was 20–25-fold in depth) (Supplemental Table S1). Lists of variants (SBSs and indels) in each sample were generated by IMR/DENOM (Gan et al. 2011).

Following mutation calling, we removed variants (versus TAIR10) that were either shared with G0 or were common between G10 plants. In further filtering, a minimum of eight and a maximum of 75 reads per site were set as the threshold limits for single base substitutions, whereas at least five reads per site were required for insertions or deletions. Following filtering, all putative variants remaining on the lists were checked by visualizing alignment files (BAM files) using Integrated Genome Viewer software (IGV; http://www.broadinstitute.org/igv). The criteria for mutation calling were set to report only variants where >95% of reads from a sample showed the same difference with respect to the G0 and to all other G10 samples. Finally, mutations that exhibited a heterozygous pattern (e.g., where 20%–80% of reads showed a base different from the reference base) or where there was insufficient coverage in G0 or G10 were also excluded from the final mutation lists. Our previous studies confirmed that ∼100% of the mutations detected by the above described multistep variant detection pipeline were real (Jiang et al. 2011; Belfield et al. 2012). Using this pipeline, we identified 102 and 52 mutations in G10 saline soil and control samples, respectively (Supplemental Tables S2, S3). Finally, standard capillary (Sanger) DNA sequencing was used to evaluate false positive mutation detection rates with respect to all mutations detected in G10-S3 and G10-S6 (21 cases). Out of 21 mutations, 19 were verified (PCR amplification failed in the remaining two cases) (Supplemental Table S9), indicating a negligible false positive detection rate.

### Calculation of Ti/Tv ratios

To calculate Ti/Tv ratios (Fig. 1D), we first determined the number of substitutions in each transition and transversion category, and then normalized these numbers by the base content (GC content 36%) of the *A. thaliana* Col-0 genome sequence. Ti/Tv ratios were then calculated as previously described (Ossowski et al. 2010; Belfield et al. 2012).

### Calculation of mutation rate

We estimate the mutation rate as follows. If *n* is the number of identified homozygous mutations per line, the mutation frequency *m* per generation is *n*/*g,* where *g* is the number of generations. Taking the average mutation rate of saline transition mutations as an example, *n* = 42 mutations/9 lines, and *g* = 10, the mutation rate *m* = 4.67/10 = 0.467. However, this calculation may be an underestimation of the real mutation rate due to the limited number of generations (Hoffman et al. 2004; Ossowski et al. 2010), for the following reasons. All new mutations are heterozygous when they arise, and one quarter of the heterozygous mutations present in the germ line before the specialization of the reproductive tissues are expected to be inherited in the homozygous state at the beginning of the next generation (Hoffman et al. 2004; Ossowski et al. 2010). Let *μ* be the probability of a new homozygous mutation per generation, and *τ* be the probability of a new heterozygous mutation per generation. It can be shown that the total probability of accumulated homozygous mutations over *g* generations is
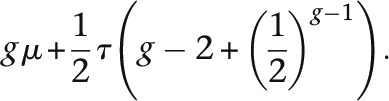
This causes the count of mutations accumulating after *g* generations to be *g*(*μ* + *τ*/2). In the present case, *g* = 10, the count of identified homozygous mutations is then 10*μ +* 8*τ*/2, which is lower than the expected count of accumulated homozygous mutations, i.e., 10 × (*μ* + *τ*/2). Although we acknowledge an underestimation of the mutation rate, current knowledge does not permit us to accurately correct it, because the values of *μ* and *τ* are hard to estimate during plant development (Hoffman et al. 2004; Ossowski et al. 2010). Nevertheless, this underestimation has a limited effect on our results and conclusions because, even in the worst-case scenario, if all mutations originated after the specialization of the reproductive tissues and none originated before the specialization of the reproductive tissues, our current estimation (8*τ*/2) would be 8/10 times the number of true accumulated mutations (10*τ*/2), thus causing a 20% underestimation of the real mutation rate. In addition, since the same approach is used to estimate the mutation rates in both control and saline samples, our analysis should have little effect when comparing mutation rates between them.

### Whole-genome bisulfite sequencing and alignment

Genomic DNA samples were sequenced using standard bisulfite sequencing protocols and Illumina 90-bp paired-end sequencing technology at BGI, China. Sequencing reads were aligned to the *Arabidopsis* reference genome (TAIR10) using Bismark alignment software v.0.7.7 (Krueger and Andrews 2011) with a maximum of two mismatches, and only uniquely aligned reads were retained. Bisulfite conversion rates (Supplemental Table S4) were estimated based on reads that uniquely aligned to the lambda phage genome.

### Identification of methylated cytosines

Methylated cytosine positions were identified from the cytosine sites reported by Bismark v.0.7.7. We began with a set of between 40,204,446 and 41,625,777 cytosine positions in each genomic sample (Supplemental Table S5), each position being represented by at least three, and at most, two hundred high-quality reads. Next, between 3,175,834 and 4,065,685 cytosine positions were found to be covered by at least three methylated reads in individual samples and were regarded as methylated sites (Supplemental Table S5).

### Identification of differentially methylated cytosine positions

To identify DMPs in G10 MA lineages, we restricted our consideration to those positions which had at least three and at most 200 reads across all G1 and G10 genomic DNA samples. Out of the 38,149,921 cytosine positions which passed the coverage threshold, 5,222,311 positions were found to be methylated in at least one G1 or G10 sample. We then analyzed these cytosine positions to identify sites displaying significant methylation differences between samples, using a modification of a previously published approach (Becker et al. 2011). We first performed Fisher’s exact test between sites and obtained *P*-values for pairwise tests between different lines. *P*-values from individual tests were adjusted for multiple comparisons with the Bonferroni correction, and then used for estimation of genome-wide false-discovery rates (FDRs) using the Benjamini and Hochberg method (Benjamini and Hochberg 1995). To reduce false positives, we first identified a total of 10,591 DMPs distinguishing the two G1 parental lines at a relaxed FDR of 10% and removed these from the list of methylated positions. The remaining 5,211,720 positions were tested for differential methylation between generations (i.e., a difference in methylation status [gain or loss of methylation] between G1 and G10 samples). We conducted 12 pairwise tests of each of the six G10 samples against the two G1 parental samples, thus identifying positions that were differentially methylated in at least one of the six G10 samples with respect to both G1 parental samples at a FDR of 5%. This analysis revealed 44,957 DMPs (the sum total of DMPs in six G10 MA samples) where methylation status differed between generations. We used a similar approach to identify DMPs in the G11-S2 and G11-C1 samples, at a FDR of 5%.

### Identification of differentially methylated cytosine regions

Using a previously reported strategy (Becker et al. 2011), we consolidated DMPs (identified as described above) on the basis of relative genomic location. DMPs were considered to be adjacent if they were located less than 50 bp apart from one another. Regions containing less than five DMPs or <10 bp were ignored. Remaining regions were then tested (using Fisher’s exact test) for differential methylation by averaging over the number of methylated and nonmethylated reads covering the DMPs constituting each region. As before, *P*-values were corrected for multiple comparisons using the Bonferroni correction, and a genome-wide FDR was estimated. A FDR of 5% was used to identify regions differentially methylated in G10 samples (versus G1 parental samples). This resulted in the identification of 14 DMRs in G10 control samples and 46 DMRs in G10 saline soil samples.

## Data access

The genomic resequencing and bisulfite sequencing data from this study have been submitted to the NCBI Sequence Read Archive (SRA; http://www.ncbi.nlm.nih.gov/sra) under accession numbers SRP045804 and SRP047267, respectively.
